# Supplementary material for: A Global Gene Body Methylation Measure Correlates Independently with Overall Survival in Solid Cancer Types
Source: Cancers (Basel). 2020 Aug 12;12(8):2257. doi: 10.3390/cancers12082257 (PMC7464642; doi:10.3390/cancers12082257)
Supplement: Supplementary file 1 [file cancers-12-02257-s001.zip › Supplement.pdf]

cancers

MDPI

Article

A Global Gene Body Methylation Measure Correlates Independently with Overall Survival in Solid Cancer Types - *Supplement*

Dietmar Pils [1,*], Elisabeth Steindl [1,†], Anna Bachmayr-Heyda [2,†,‡], Sabine Dekan [3] and Stefanie Aust [2]

[1] Division of General Surgery, Department of Surgery, Comprehensive Cancer Center (CCC) Vienna, Medical University of Vienna, 1090 Vienna, Austria; Elisabeth.Steindl@meduniwien.ac.at

[2] Department of Obstetrics and Gynecology, Comprehensive Cancer Center (CCC) Vienna, Medical University of Vienna, 1090 Vienna, Austria; Anna.Bachmayr@hotmail.com (A.B.H.); Stefanie.Aust@meduniwien.ac.at (S.A.)

[3] Department of Pathology, Medical University of Vienna, 1090 Vienna, Austria; Sabine.Dekan@meduniwien.ac.at

* Correspondence: dietmar.pils@univie.ac.at; Tel: +0043 1 40400 41690; Fax: +0043 1 40400 66740

† These authors contributed equally.

‡ Current address: Research Cancer Immunology, Boehringer Ingelheim RCV GmbH & Co KG, Dr. Boehringer Gasse 5-11, 1120 Vienna, Austria

cancers

MDPI

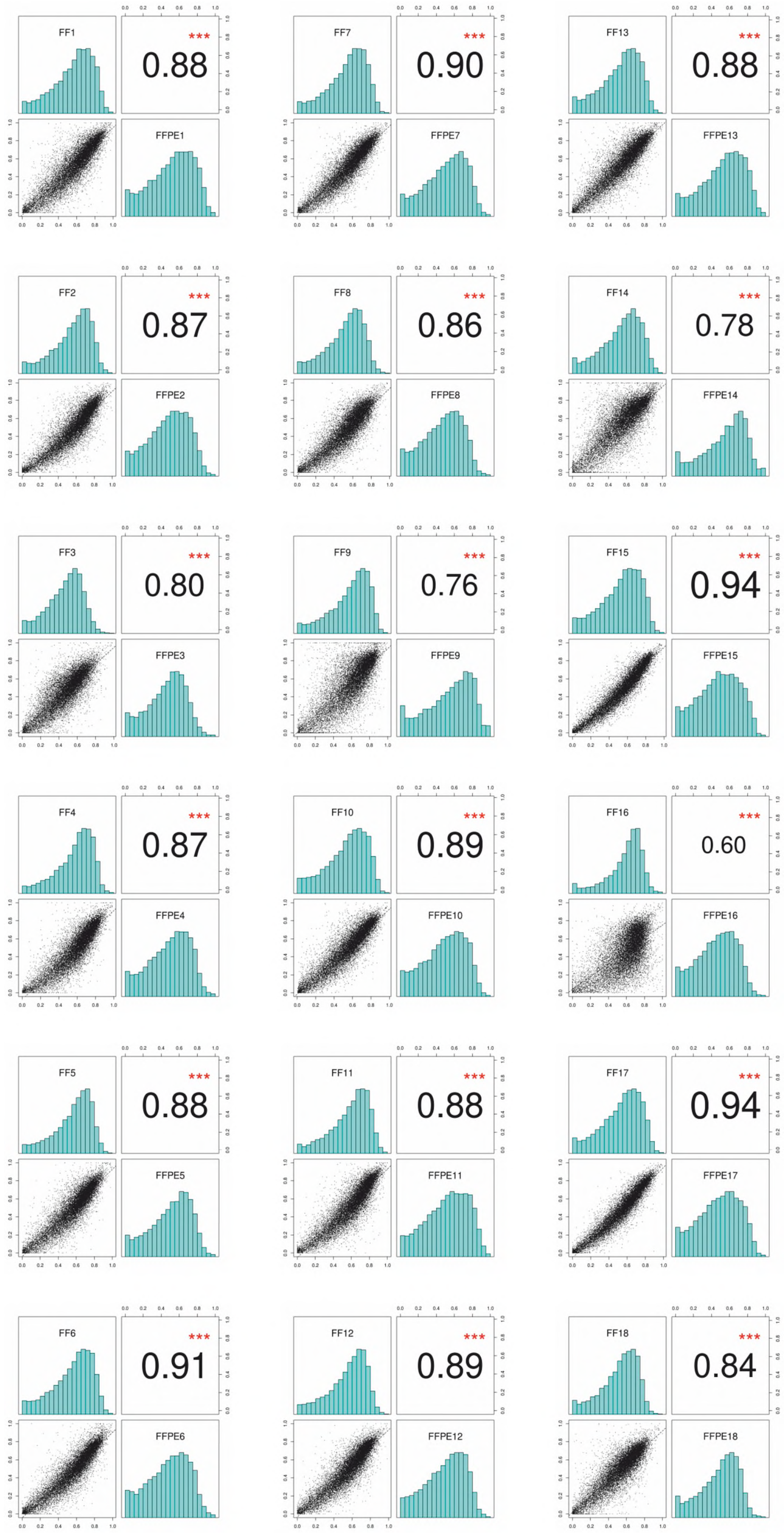

Figure S1. Correlation plots of averaged GB methylation values between DNA isolated from fresh frozen tumor tissues and macro/microdissected FFPE tumor tissues of the same patients.

cancers

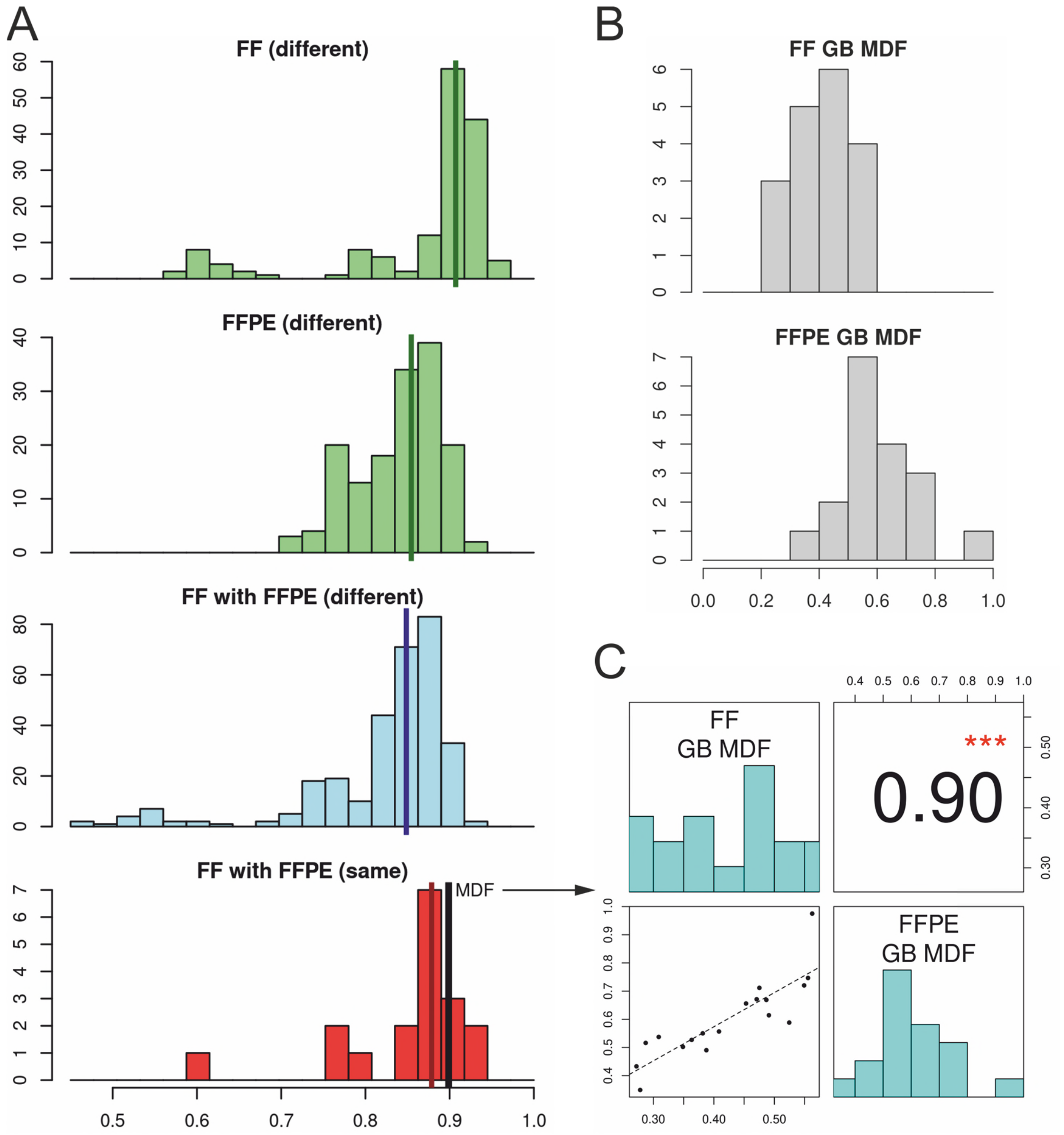

Figure S2. **(A)** Histograms of correlation coefficients of averaged GB methylation values between different samples within (fresh frozen) FF and (formalin fixed and paraffin embedded) FFPE tissues (green color), between different samples across FF and FFPE sample (blue color) and between the matched pairs of samples (from the same patients) from FF and FFPE tissues (red color). The black line indicates the correlation coefficient of the MDF values between matched pairs of FF and FFPE tissues (*cf.* C). **(B)** Histograms of the calculated "Methylation Definition Factor" (MDF) from FF and FFPE tissues from the same patients and **(C)** the correlation between these MDF values of the matched pairs of FF and FFPE tissues.

cancers

MDPI

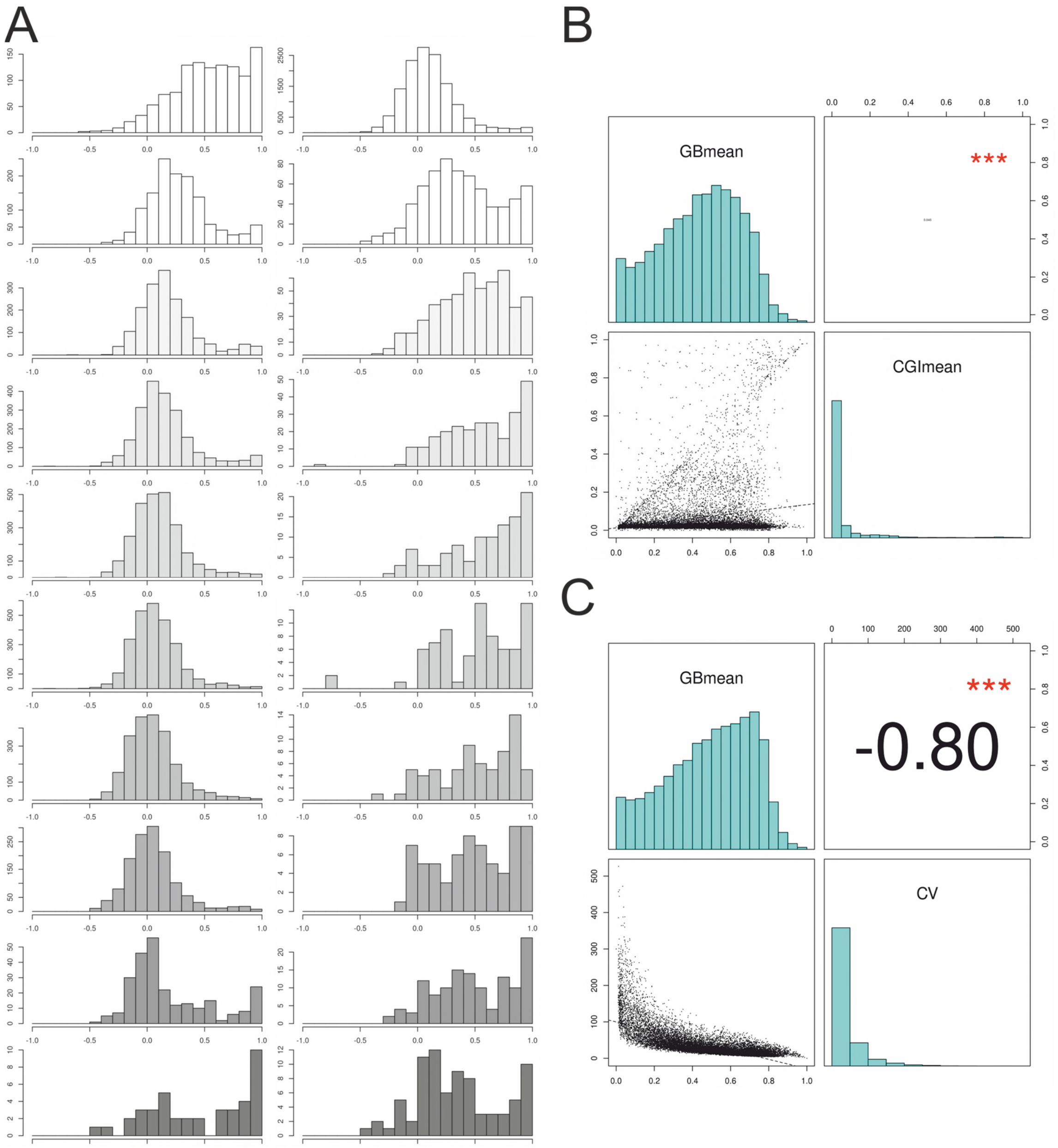

Figure S3. **(A)** Histograms of correlation coefficients of averaged GB methylation values with corresponding averaged CGI methylation values, split into slots of low to high mean methylation levels, *i.e.* 0%-10%, 10%-20%, ..., 90%-100% (colored white to dark grey). On the left side, split according GB methylation levels and of the right side according CGI methylation levels. **(B)** Overall correlation of corresponding averaged GB and CGI methylation levels. **(C)** Association of the coefficient of variation (CV) with the averaged GB methylation level.

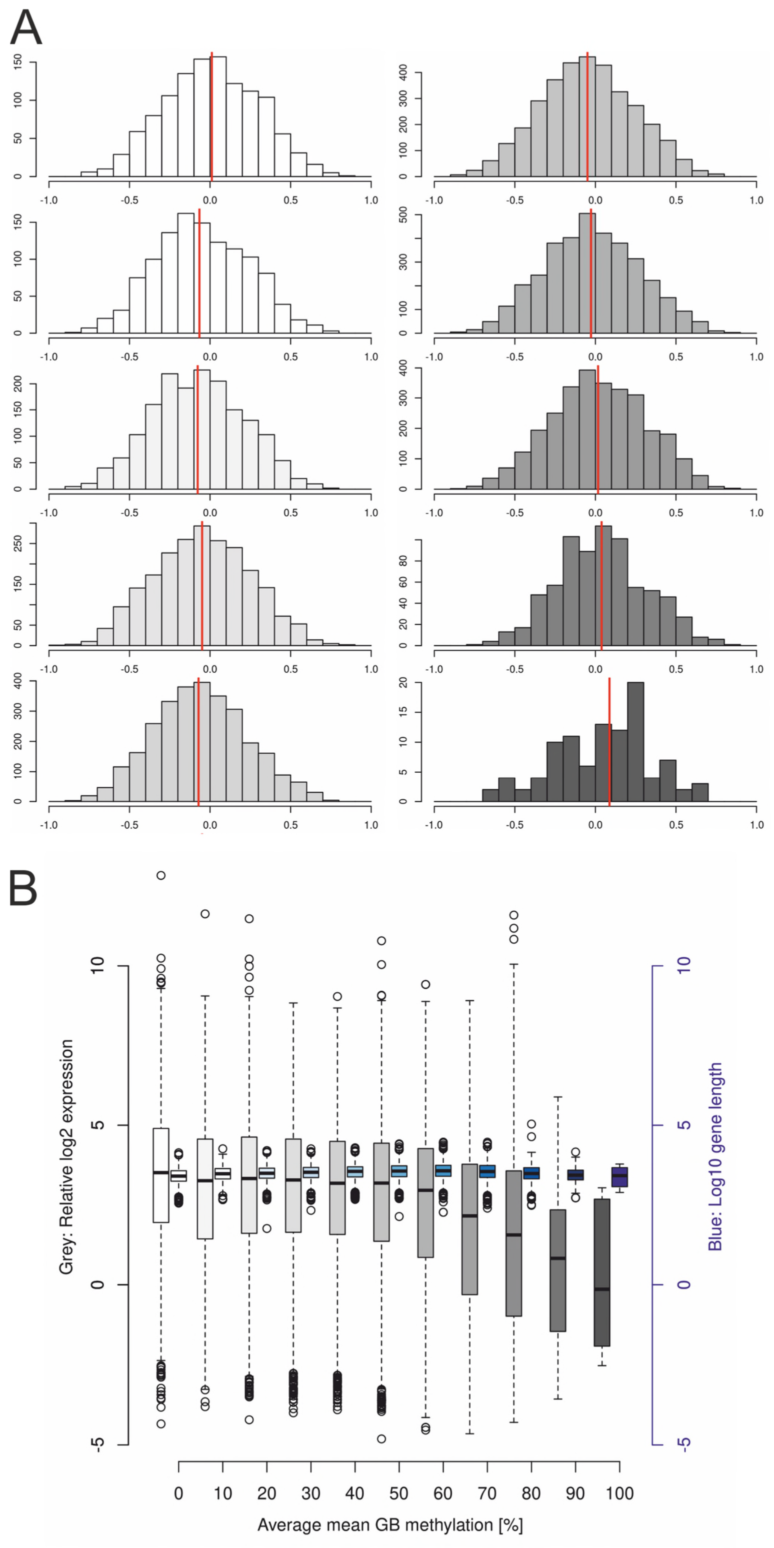

Figure S4. **(A)** Histograms of correlation coefficients between GB methylation values and corresponding gene expression values, split into slots of low to high mean GB methylation levels, *i.e.* 0%-10%, 10%-20%, ..., 90%-100% (colored white to dark grey). **(B)** Boxplots of log_2 expression values of genes in slots of 0%-10%, 10%-20%, ..., 90%-100% methylated GBs (different grey colors, left y-axis) and corresponding log_{10} gene length of genes in these slots (different blue colors, right y-axis).

cancers

MDPI

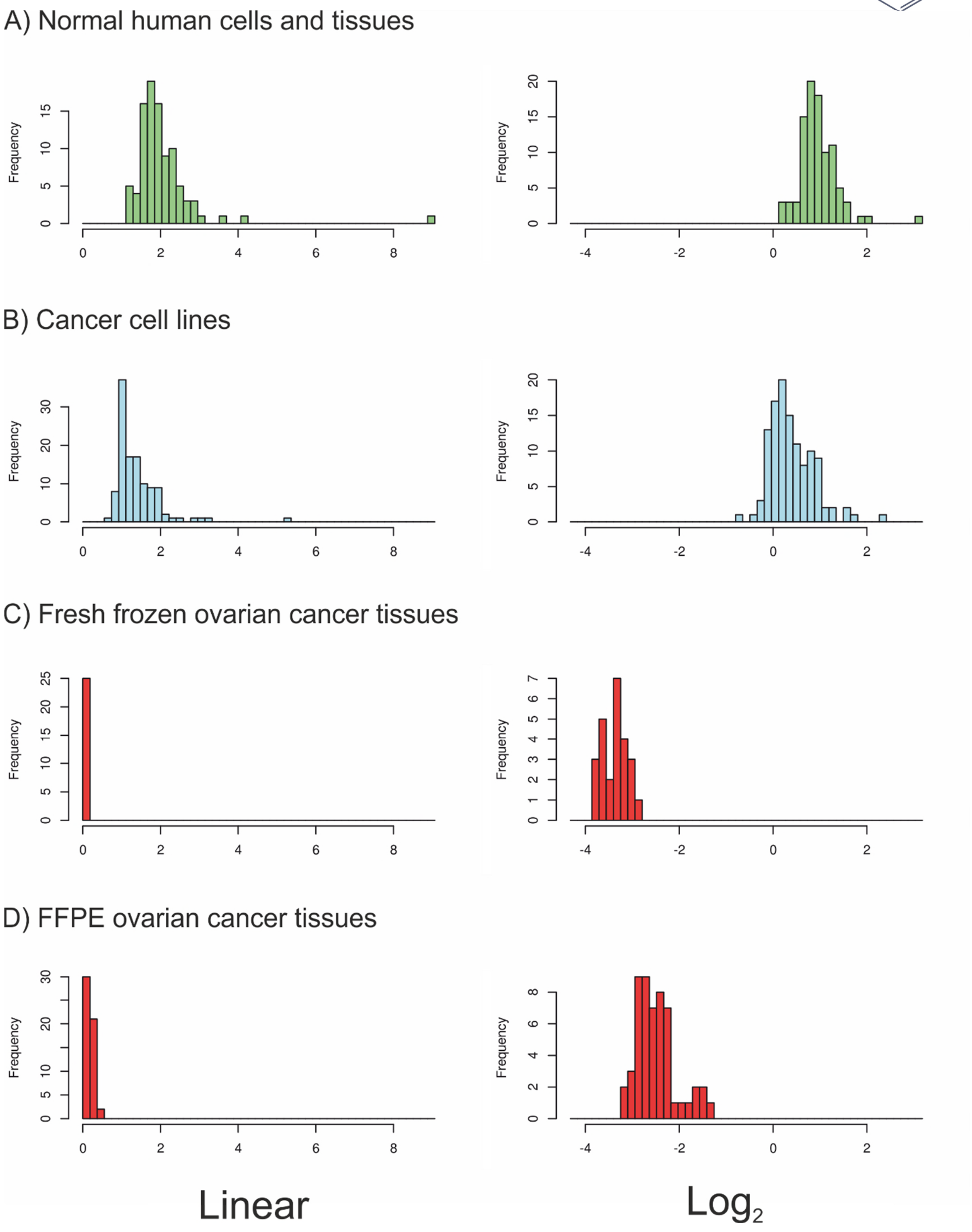

cancers

MDPI

Figure S5. Histograms of methylation definition factors (MDF, calculated as follows: (no average GB betas <0.1 + no average GB betas >0.9) / no average GB betas between 0.2 and 0.8) of **(A)** 41 normal human primary cells and tissues (astrocyte (primary cell), foreskin fibroblast (primary cell), islet of Langerhans (tissue), placenta (tissue), hepatocyte (primary cell), mononuclear cell (primary cell), testis (tissue), adrenal gland (tissue), zone of skin (tissue), mammary epithelial cell (primary cell), uterus (tissue), non-pigmented ciliary epithelial cell (primary cell), epithelial cell of alveolus of lung (primary cell), iris pigment epithelial cell (primary cell), epithelial cell of esophagus (primary cell), epithelial cell of proximal tubule (primary cell), pericardium (tissue), heart left ventricle (tissue), bronchial epithelial cell (primary cell), kidney epithelial cell (primary cell), lung (tissue), renal cortical epithelial cell (primary cell), epithelial cell of prostate (primary cell), kidney (tissue), cardiac fibroblast (primary cell), skeletal muscle cell (primary cell), stomach (tissue), epidermal melanocyte (primary cell), myoblast (primary cell), pancreas (tissue), skeletal muscle myoblast (primary cell), aortic smooth muscle cell (primary cell), osteoblast (primary cell), cardiac muscle cell (primary cell), brain (tissue), skeletal muscle tissue (tissue), choroid plexus epithelial cell (primary cell), liver (tissue), retinal pigment epithelial cell (primary cell), amniotic epithelial cell (primary cell), breast (tissue)); **(B)** 43 cancer cell lines (MCF, 10A, LNCaP, K562, IMR-90, MCF-7, HEK293, H1, HTR-8/SVneo, NB4, U-87, MG, BJ, HepG2, Caco-2, HeLa-S3, GM19240, HL-60, BE2C, T47D, CMK, Panc1, A549, GM19239, AG09319, NT2/D1, GM06990, HCT116, SK-N-SH, GM12891, SK-N-MC, AG04450, Ishikawa, AG10803, Jurkat, AG08470, AG09309, GM12878, UCH-1, PFSK-1, OVCAR3, HGPS, GM12892, AG04449, hTERT-HM); **(C)** fresh frozen ovarian cancer tumor tissues; and **(D)** formalin fixed paraffin embedded (FFPE) ovarian cancer tumor tissues.

cancers

MDPI

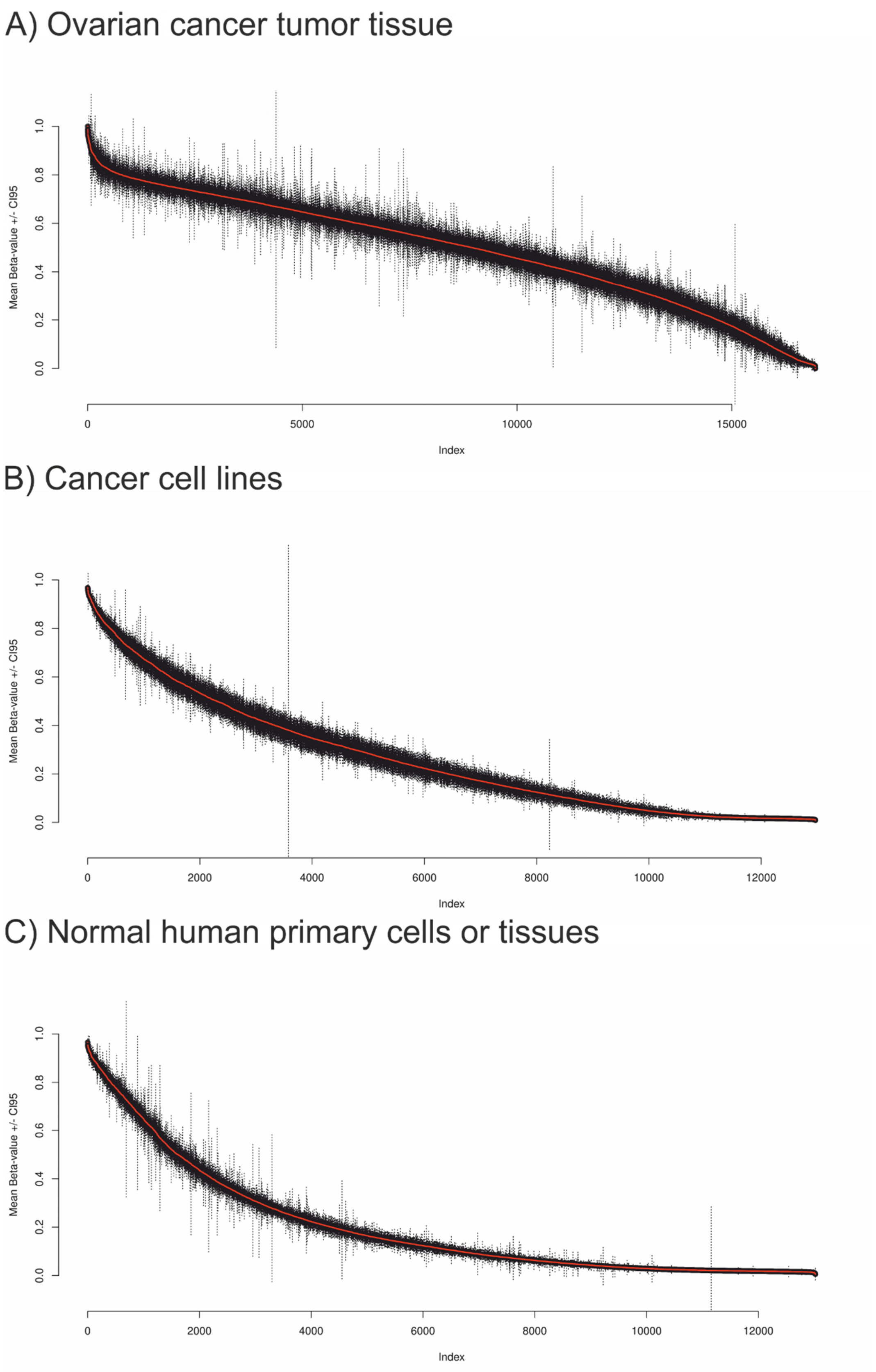

cancers MDPI

Figure S6. Distribution of average gene body methylation values (beta values ±CI_{95} over samples) of gene bodies of protein coding genes. **(A)** 45 ovarian cancer tumor tissues; **(B)** 43 cancer cell lines (MCF, 10A, LNCaP, K562, IMR-90, MCF-7, HEK293, H1, HTR-8/SVneo, NB4, U-87, MG, BJ, HepG2, Caco-2, HeLa-S3, GM19240, HL-60, BE2C, T47D, CMK, Panc1, A549, GM19239, AG09319, NT2/D1, GM06990, HCT116, SK-N-SH, GM12891, SK-N-MC, AG04450, Ishikawa, AG10803, Jurkat, AG08470, AG09309, GM12878, UCH-1, PFSK-1, OVCAR3, HGPS, GM12892, AG04449, hTERT-HM); **(C)** 41 normal human primary cells and tissues (astrocyte (primary cell), foreskin fibroblast (primary cell), islet of Langerhans (tissue), placenta (tissue), hepatocyte (primary cell), mononuclear cell (primary cell), testis (tissue), adrenal gland (tissue), zone of skin (tissue), mammary epithelial cell (primary cell), uterus (tissue), non-pigmented ciliary epithelial cell (primary cell), epithelial cell of alveolus of lung (primary cell), iris pigment epithelial cell (primary cell), epithelial cell of esophagus (primary cell), epithelial cell of proximal tubule (primary cell), pericardium (tissue), heart left ventricle (tissue), bronchial epithelial cell (primary cell), kidney epithelial cell (primary cell), lung (tissue), renal cortical epithelial cell (primary cell), epithelial cell of prostate (primary cell), kidney (tissue), cardiac fibroblast (primary cell), skeletal muscle cell (primary cell), stomach (tissue), epidermal melanocyte (primary cell), myoblast (primary cell), pancreas (tissue), skeletal muscle myoblast (primary cell), aortic smooth muscle cell (primary cell), osteoblast (primary cell), cardiac muscle cell (primary cell), brain (tissue), skeletal muscle tissue (tissue), choroid plexus epithelial cell (primary cell), liver (tissue), retinal pigment epithelial cell (primary cell), amniotic epithelial cell (primary cell), breast (tissue)).

cancers

MDPI

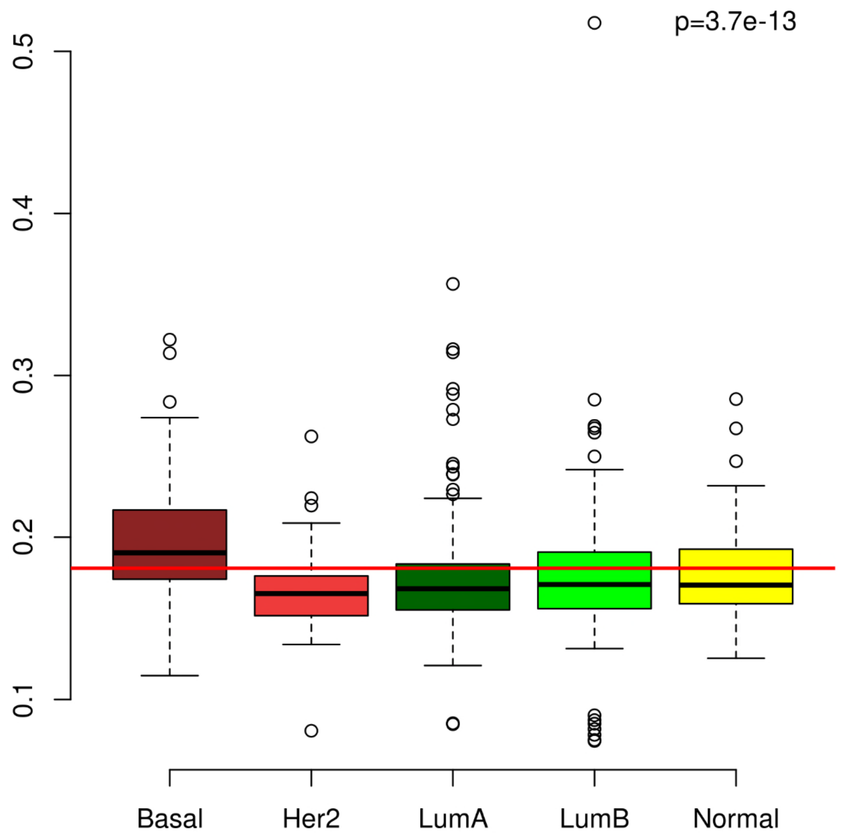

Figure S7. Correlation of the methylation definition factor (MDF), Y-axis, with the breast cancer subtypes, determined with the PAM50 gene signature. Red line, cutoff for the optimal dichotomization of the MDF as obtained from multiple Cox regression models.

cancers

MDPI

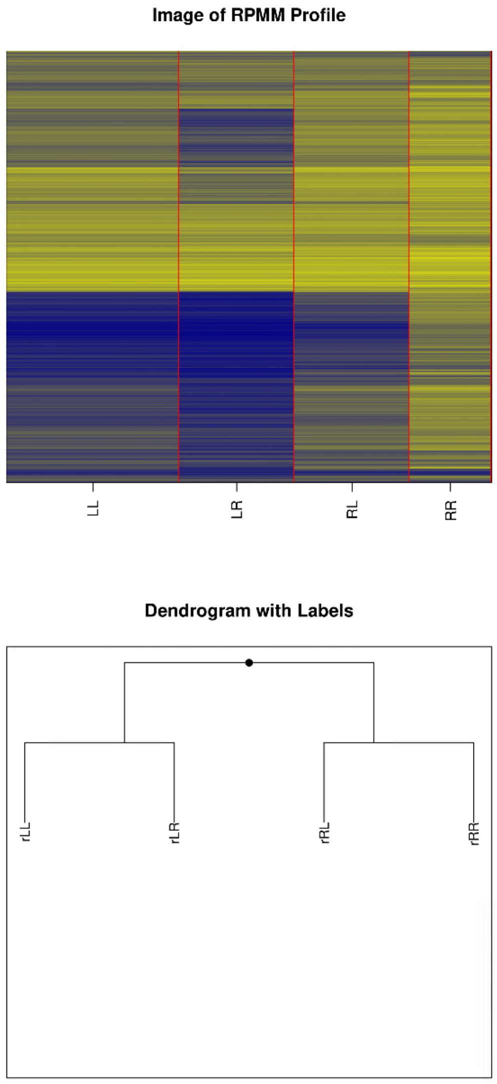

Figure S8. The CpG island methylator phenotype (CIMP) status determined from TCGA colorectal cancer samples according to Hinoue *et al*. [1]. rRR, CIMP-H; rRL, CIMP-L; rLL, type 3; rLR, type 4.

cancers

MDPI

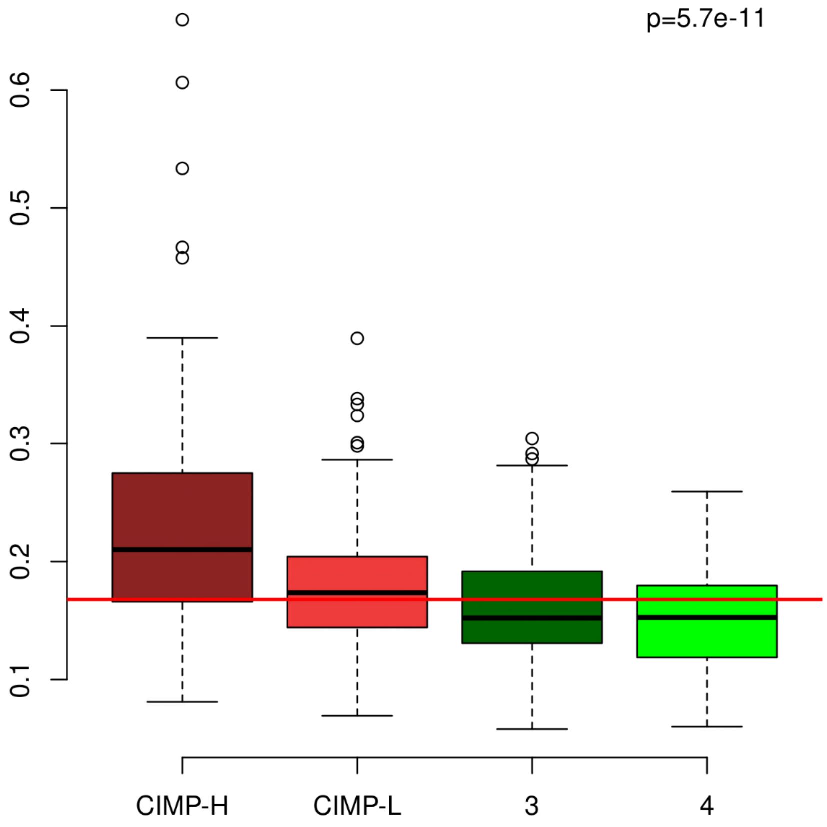

Figure S9. Correlation of the methylation definition factor (MDF), Y-axis, with the CpG Island Methylator Phenotypes determined with the function bclTree of R-package RPMM 1.25. Red line, cutoff for the optimal dichotomization of the MDF as obtained from multiple Cox regression models.

cancers

MDPI

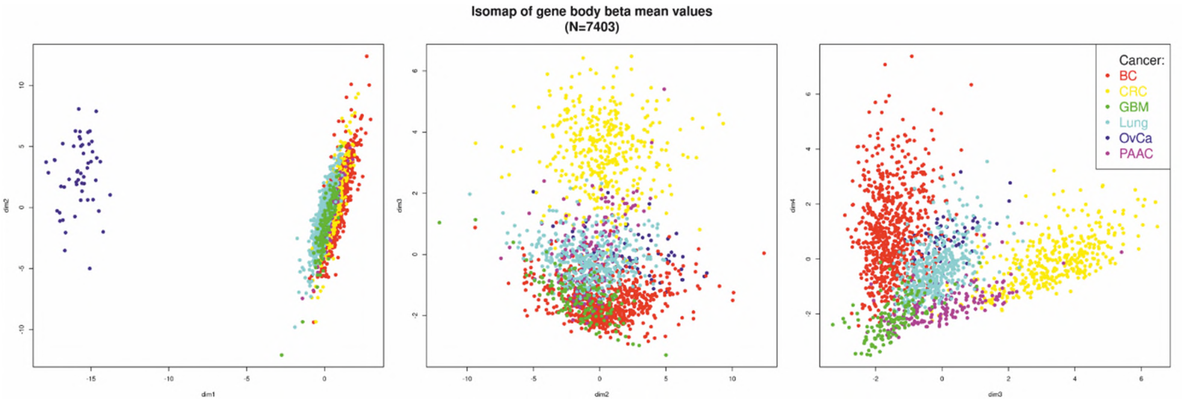

Figure S10. Isomap representation of all samples from all cancer entities using the averaged gene body beta values (if present and without missing data in all samples, N=7,403).

cancers

MDPI

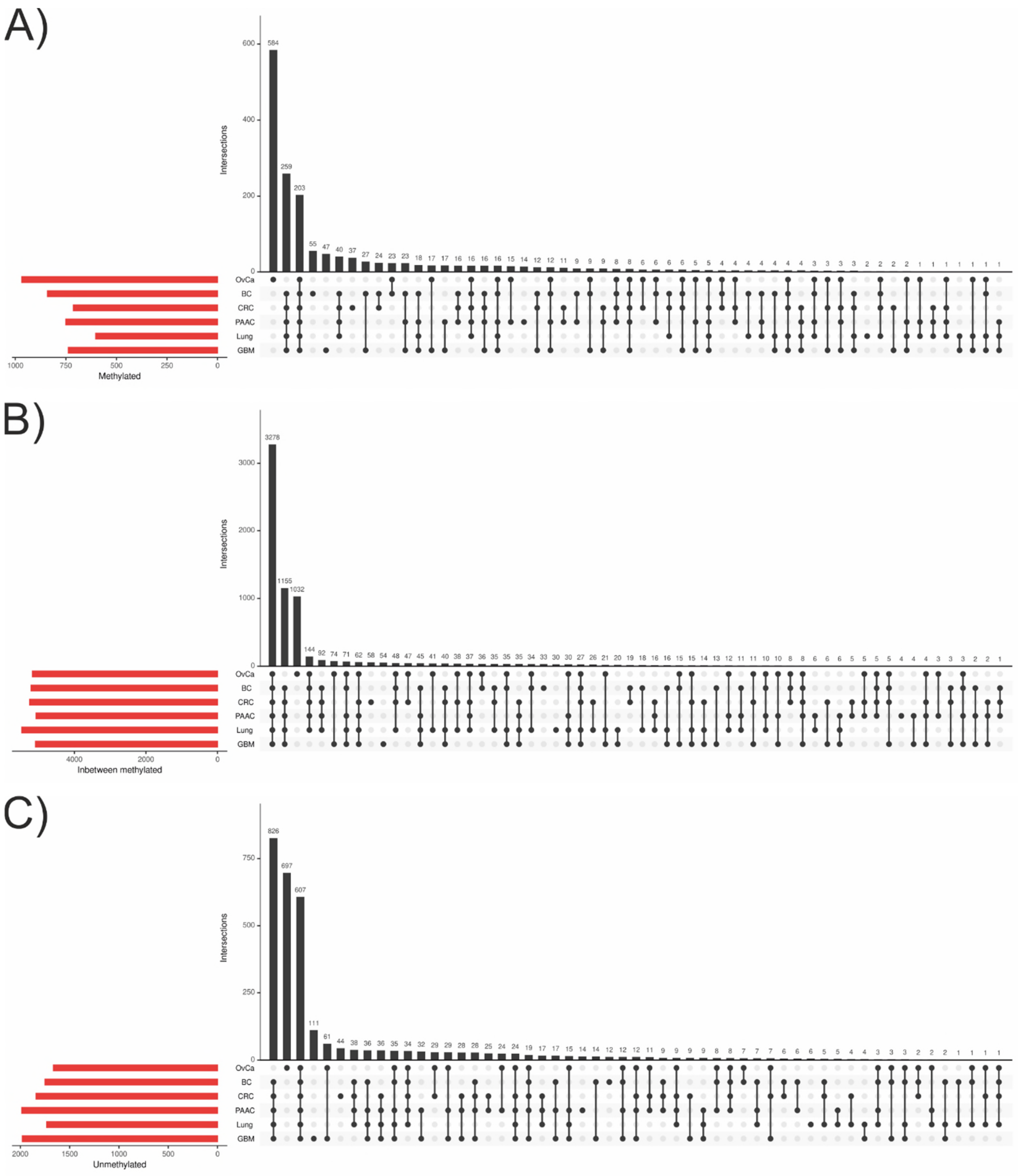

Figure S11. Overlap of **(A)** genes with methylated gene bodies (mean averaged beta values >0.7), **(B)** with intermediate methylated gene bodies (mean averaged beta values <0.7 and >0.3), and **(C)** unmethylated (mean averaged beta value <0.3) over all indicated cancer types. Only gene bodies with available averaged beta values present in all cancer entities were considered; N=7,801.

cancers

MDPI

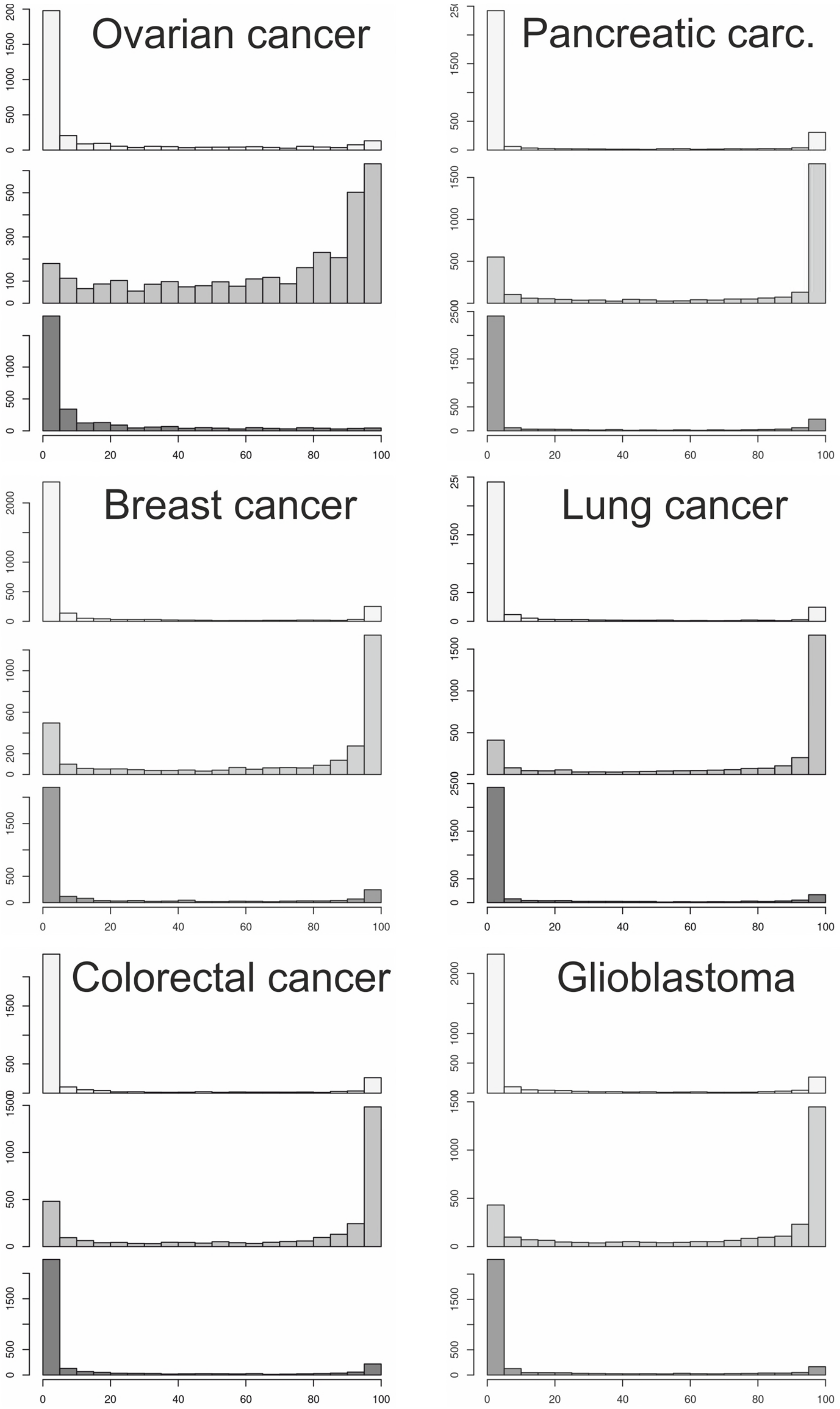

Figure S12. Histograms showing the percentages of genes which are consistently annotated to a specific GB methylation slot, divided into three mean GB methylation slots, low methylated (<0.3; light grey), intermediate methylated (≥0.3 and ≤0.7; grey), and high methylated (>0.7; dark grey).

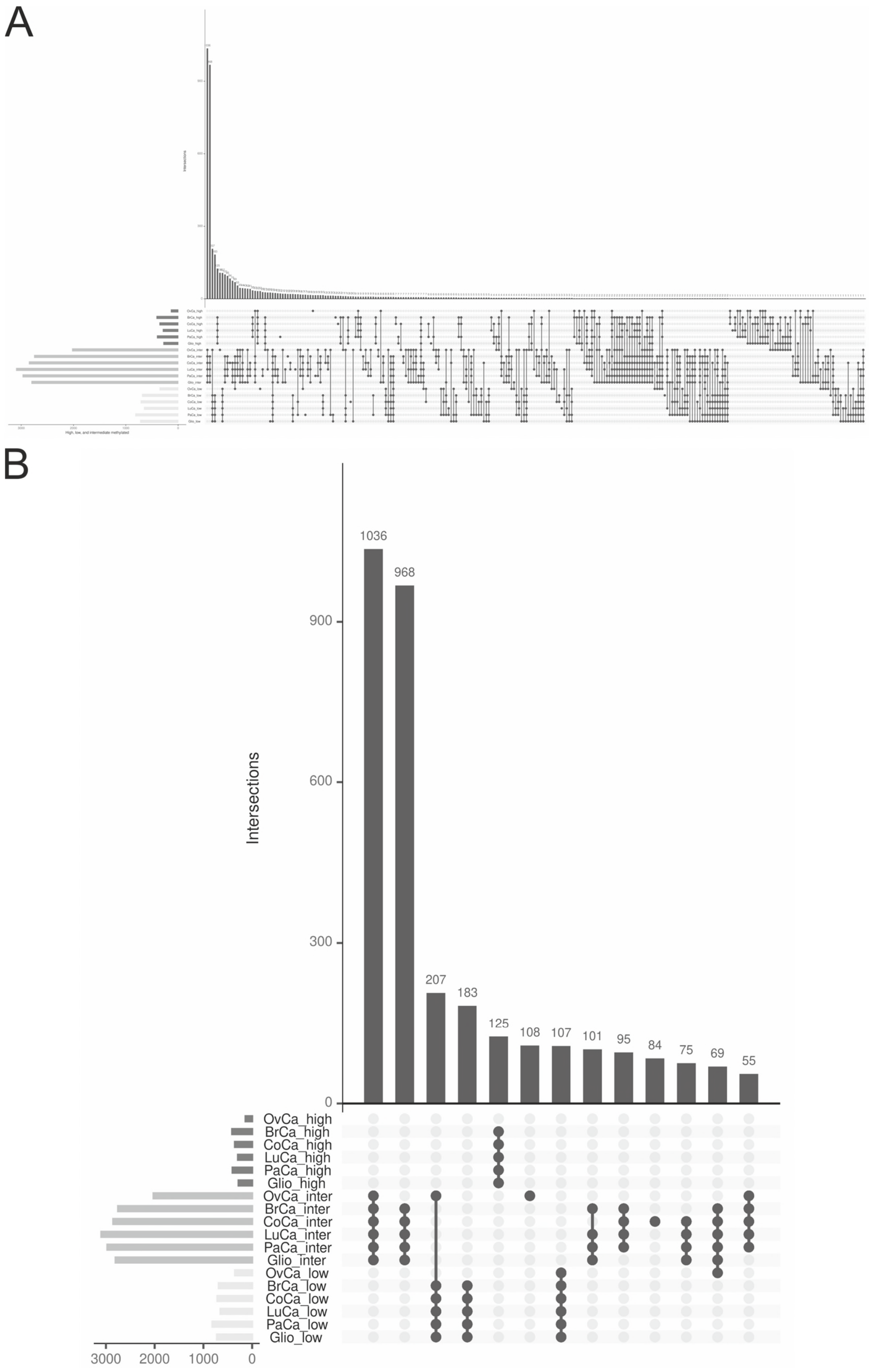

Figure S13. (A) Overlap of gene lists consistently categorized (*i.e.* in >90% of samples) to one of the GB methylation slots (high, >0.7; intermediate; and low, <0.3) from different cancer entities, derived from data shown in Figure S12. **(B)** Subset of the first 13 gene overlaps from A).

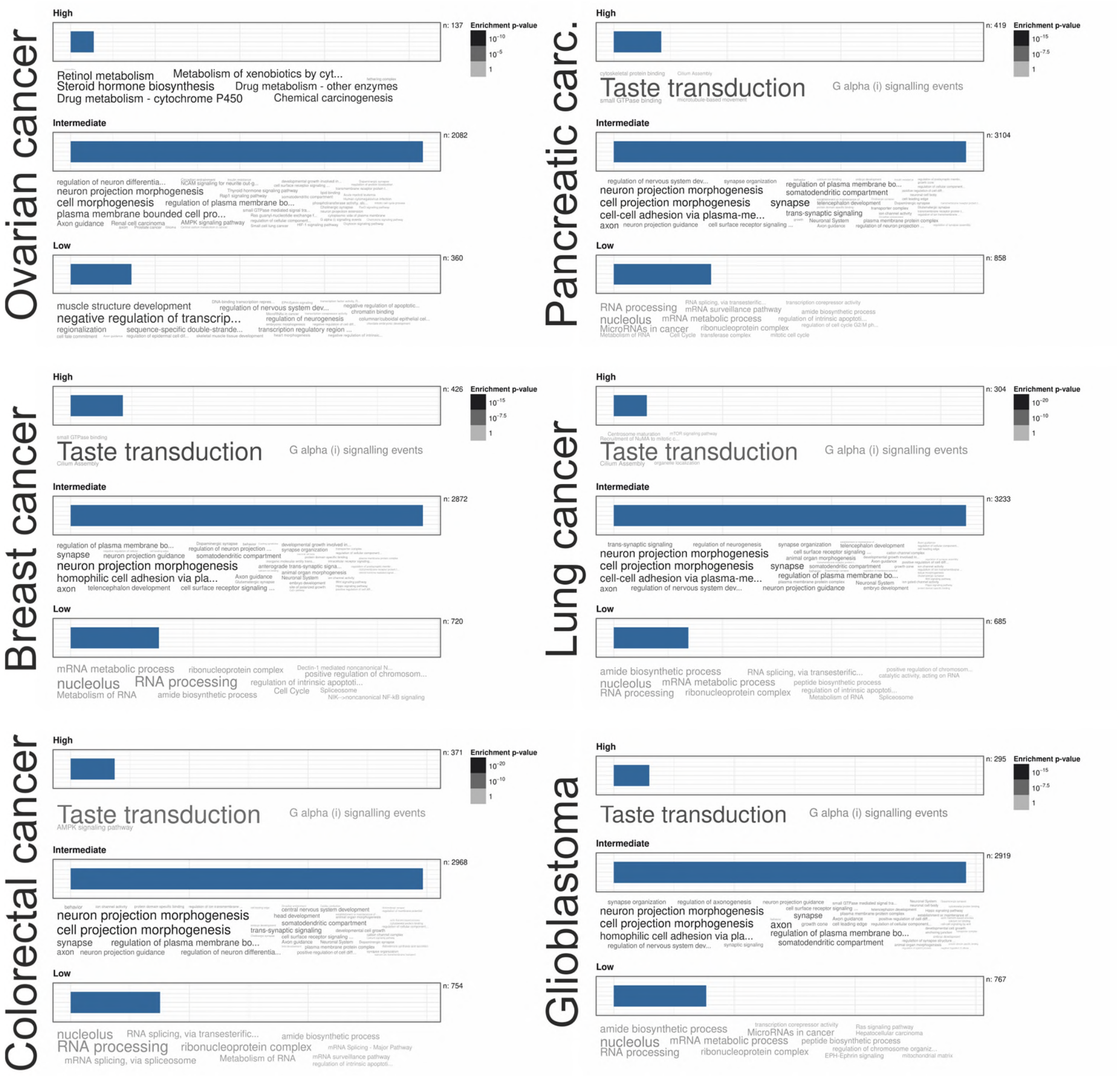

Figure S14. Enrichment plots of gene ontology terms and pathways of lists of consistently as high, intermediate, or low methylated categorized genes. Enrichments are indicated in word clouds by size and color (p-values). Overlap of gene lists across cancer entities is shown in Figure S13.

cancers

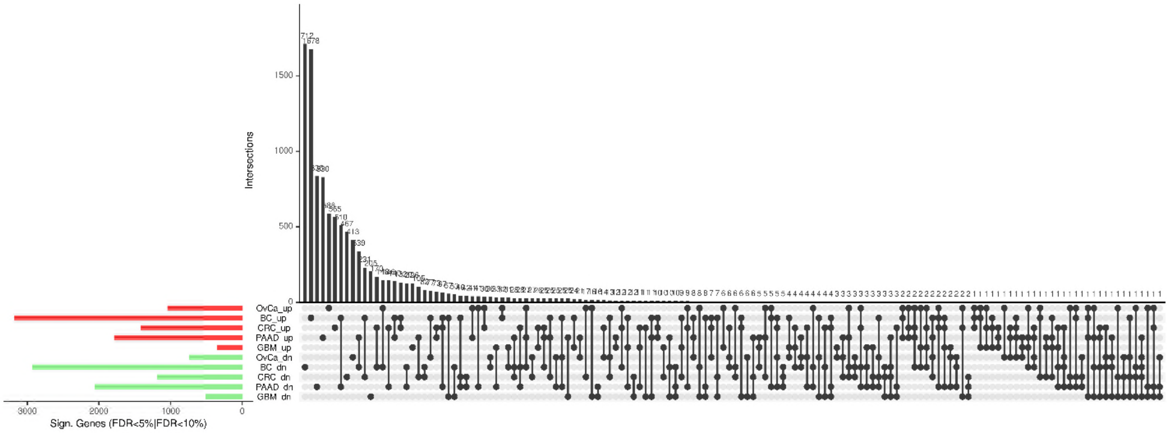

Figure S15. Overlap of genes positively (red) or negatively (green) correlated to the respective MDF/MUMF in different cancer entities. For OvCa and GBM a 10% FDR cutoff, for all other cancer entities, a 5% FDR cutoff was used.

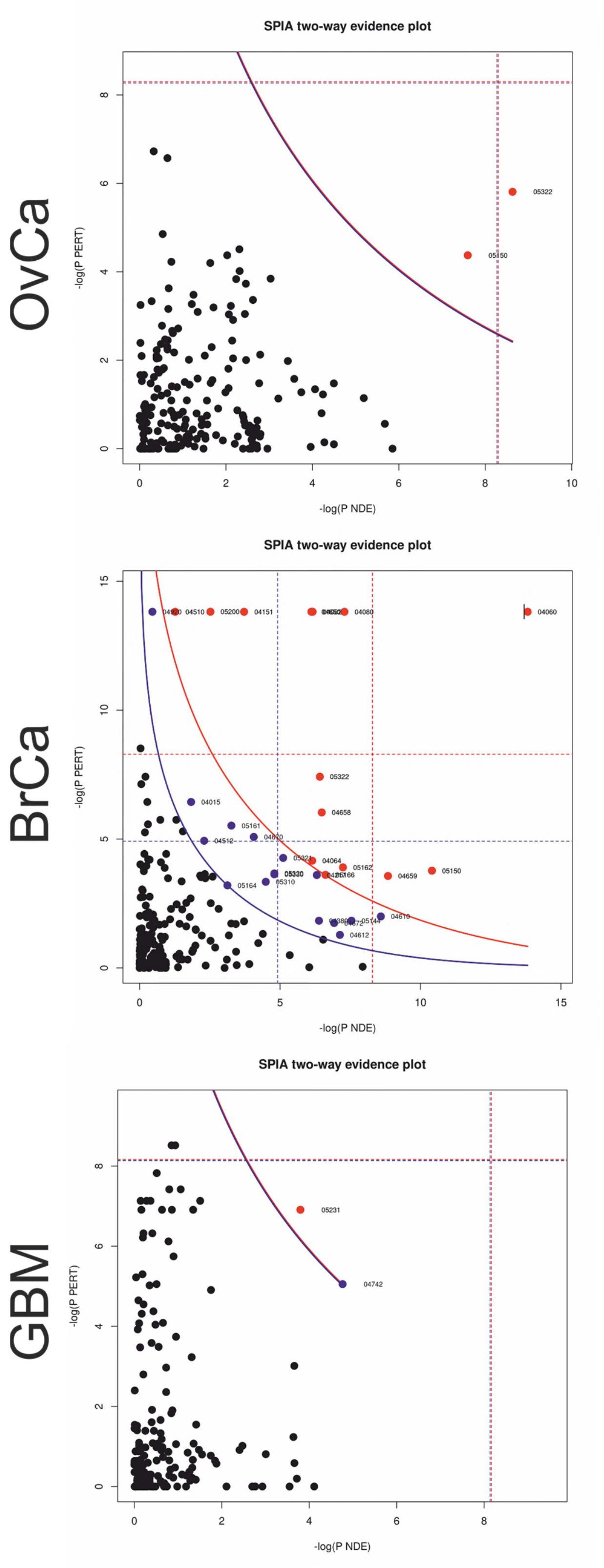

Figure S16. SPIA evidence plot of significantly deregulated KEGG pathways, blue and red dots with KEGG pathway numbers (blue line: cutoff FDR <5%). For colorectal cancer and pancreatic carcinoma no significant pathways were revealed (*cf*. Tables S1).

cancers

MDPI

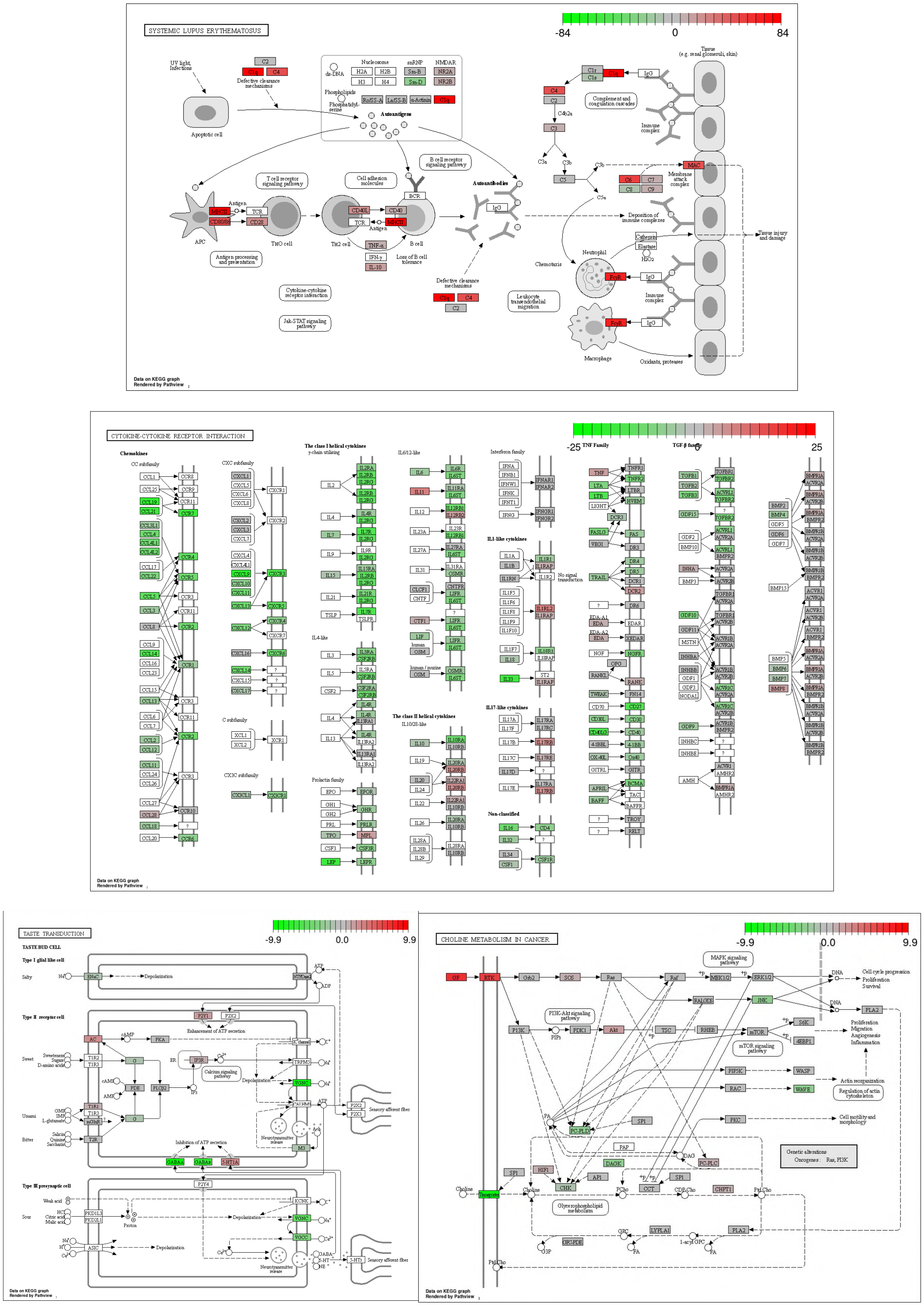

Figure S17. Most significant KEGG pathways associated with the Methylation Definition Factor (MDF) in ovarian and breast cancer or the Methylation-over-Unmethylation Factor (MUMF) for glioblastoma. **(A)** Inhibited pathway "Systemic Lupus Erythematosus" in ovarian cancer. **(B)** Inhibited pathway "Cytokine-Cytokine Receptor Interaction" in breast cancer, and **(C)** two identical significant pathways, one inhibited. "Taste Transduction" and one activated, "Choline Metabolism in Cancer" in glioblastoma. Green-to-red color bar represents log_2 fold changes correlated to the MDF or MUMF.

cancers

1. Hinoue, T.; Weisenberger, D.J.; Lange, C.P.; Shen, H.; Byun, H.M.; Van Den Berg, D.; Malik, S.; Pan, F.; Noushmehr, H.; van Dijk, C.M., et al. Genome-scale analysis of aberrant DNA methylation in colorectal cancer. *Genome Res* **2012**, *22*, 271-282, doi:10.1101/gr.117523.110.

© 2020 by the authors. Licensee MDPI, Basel, Switzerland. This article is an open access article distributed under the terms and conditions of the Creative Commons Attribution (CC BY) license (http://creativecommons.org/licenses/by/4.0/).
